# Dosimetric evaluation of a newly designed low dose rate brachytherapy applicator for treatment of cervical cancer with extension into the lower vagina

**DOI:** 10.1120/jacmp.v8i2.2400

**Published:** 2007-04-19

**Authors:** Curtis Baker, Sharifeh A. Dini, Mahesh Kudrimoti, Shahid B. Awan, Ali S. Meigooni

**Affiliations:** ^1^ University of Kentucky Chandler Medical Center Department of Radiation Medicine Lexington Kentucky U.S.A.

**Keywords:** cervical cancer, dosimetry, vaginal, Meigooni applicator

## Abstract

Currently, patients having cervical cancer with extension into the lower vagina are being treated with a combination of the Fletcher–Suit applicator, which treats the cervix, and a vaginal cylinder, which treats the lower vagina. With this method, patients receive two separate implants—a procedure that creates greater uncertainty in the dose distribution and unnecessary patient inconvenience.

To reduce the uncertainty of the dose delivery and to eliminate patient inconvenience, a new applicator was designed and fabricated at the University of Kentucky for treatment of cervical cancer extending into the lower vagina. In addition, the geometric design of the new device allows for treatment of cervical cancer without extension into the lower vagina and simultaneously provides advantages relative to the commonly used Fletcher–Suit applicator.

The dosimetric characteristics of this new applicator (hereafter called Meigooni applicator) were determined using experimental procedures. The measurements were performed using tissue‐equivalent phantom material (Solid Water: Gammex RMI, Middleton, WI) that was machined to accommodate the applicator and LiF thermoluminescent dosimetry chips. The applicator was loaded with C137s brachytherapy sources in a standard loading scheme. A similar experimental procedure was performed using the currently available Fletcher–Suit mini‐ovoid applicator. The results obtained with each applicator were compared with the values calculated by two commercially available treatment planning systems.

The experiments showed that the Meigooni applicator allows for safe single treatment of cervical cancer that has extended into the lower vagina, eliminating the need for two separate treatment techniques. Moreover, the Meigooni applicator can function as an alternative to the Fletcher–Suit applicator for the treatment of patients with cervical cancer.

PACS number: 87.53.Jw

## I. INTRODUCTION

Recently, much debate has arisen over the use of high‐dose‐rate (HDR) versus low‐dose‐rate (LDR) brachytherapy procedures. After reviewing various intracavitary procedures, Brenner and Hall noted that physicians now have the choice of using HDR as opposed to the traditional LDR for brachytherapy implants.^(1)^ Although faster treatments and shorter hospital visits are certainly advantages of the HDR treatment modality, the question of which treatment method provides the best tumor control and the fewest side effects is still uncertain.^(2)^ However, Hareyama et al.^(3)^ showed that patients treated with LDR (as compared with HDR) brachytherapy for stage II cervical cancer had a better overall 5‐year survival rate.

As diagnoses of cervical cancer continue to rise, physicians and physicists must remain current with the rapidly changing field of radiation oncology so as to provide patients with the best possible treatment. In 2005, 12,800 new cases of cervical cancer were reported, with a mortality rate of one third, indicating a need for continuing research and new modalities.^(4)^

Many patients diagnosed with cervical cancer undergo a standard treatment regimen of 45 Gy to the pelvis delivered by external‐beam radiation, followed by brachytherapy implants, and finally an external‐beam parametrial boost.^(5)^ Currently, cervical cancer patients are treated using either HDR or LDR brachytherapy. The physician must decide on the implant and dose rate that will achieve the best isodose distribution while limiting the early and late effects of radiation.

In a recent study by Ferrigno et al., HDR and LDR brachytherapy treatments were compared.^(6)^ Those authors found that overall 5‐year survival and locoregional control were both increased with LDR brachytherapy for cervical cancer.

Irregular tumor volume and size (small or large) has been a major limitation in LDR brachytherapy.^(7)^ The stage of this gynecologic cancer, together with the tumor extent, plays a major role in choice of the proper applicator. One common treatment device is the Fletcher– Suit applicator, more commonly known as “tandem and ovoid” (T&O). However, this device does not provide sufficient dose distribution to treat cervical cancer that has extended into the lower vagina. One common practice is to use the Fletcher–Suit applicator to treat the cervical portion of the disease, and to follow with an implant, using a vaginal cylinder to treat the lower vagina. However, providing accurate dose distributions by matching these two separate treatments is difficult. In addition, the treatment procedure is costly and inconvenient for the patient.

To resolve the foregoing problems, a new applicator—hereinafter called the Meigooni applicator—was designed to treat the entire region at one time. The design of the new device also permits it to be used as an alternative to the Fletcher–Suit applicator for treatment of patients with cervical cancer without extension to the lower vagina.

The goal of the present work was to determine the dosimetric characteristics of the Meigooni applicator and to review that applicator as an alternative to the standard T&O in the treatment of cervical cancer.

## II. MATERIALS AND METHODS

### A. Meigooni applicator description and design

**Figure 1:**
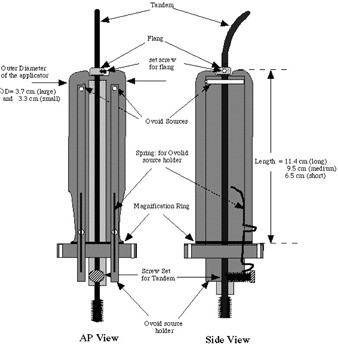
A schematic diagram of the Meigooni applicator showing the anterior and lateral views. AP = anterior–posterior.

Fig. 1 shows a schematic diagram of the Meigooni applicator. The applicator consists of a polystyrene cylinder housing a steel tandem that slides through the center of the applicator. Two rectangular ovoids (also made of polystyrene) are constructed so that they slide into the cylinder and rest side by side with approximately 1.2 cm of space between the sources. At each end of the ovoids, a drilled cylindrical hole holds a single C137s brachytherapy source. At the base of the applicator, a polystyrene screw holds the tandem in place after accurate measurements of the patient's cervix have been made. Currently, the Meigooni applicator is available in three lengths (6 cm, 9 cm, 12 cm) and three diameters (3.1 cm, 3.3 cm, 3.7 cm).

### B. Intended use

A primary function of the Meigooni applicator is to treat cervical cancer that has extended into the lower vagina. To accomplish this goal, the sources are extended down through the cylinder, past the commonly prescribed active length of 6 cm that is frequently used for cervical cancer treatments with a Fletcher–Suit T&O applicator.

**Figure 2:**
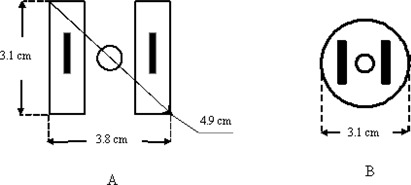
A schematic diagram of the cross‐sectional view of (A) the Fletcher–Suit mini‐ovoid applicator and (B) the Meigooni applicator. (Drawings not to scale.)

As shown in Fig. 2, the smallest dimension of the Fletcher–Suit applicator with mini‐ovoid is approximately 3.8 cm. The Meigooni applicator, on the other hand, is able to accommodate patients with smaller dimensions (3.1 cm) and is easily inserted owing to its reduced diameter and cylindrical design.

The common prescription point for cervical cancer is point A—the intersection of the uterine artery and the ureter. One objective of the present work was to assess the dose to that point. Also, the dose to points inside the vaginal wall and to the bladder and rectum were calculated. Using Solid Water (Gammex RMI, Middleton, WI) phantoms and thermoluminescent dosimeters (TLDs), a simulated patient was created in which to perform dose calculations for the points of interest.

### C. Experimental setup for dose measurement

**Figure 3:**
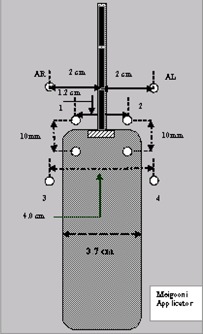
A schematic diagram of the experimental setup for thermoluminescent dosimetry (round symbols) with the Meigooni applicator. In this figure, point A‐right (AR) and point A‐left (AL) are both clearly identified.

The Meigooni applicator chosen for the present experiment was 3.7 cm in diameter and 12 cm in length, and had 1.2‐cm spacing between the ovoids. Two pieces of tissue‐equivalent material (Solid Water) were designed to hold the applicator and TLD chips in the desired locations. Two Solid Water slabs, each 30×30×5 cm, were machined to hold the applicator between them with minimal tolerance. Eight holes (2‐mm diameter, 1‐mm depth) corresponding to the dose calculation points, were drilled into the bottom slab. Each prescription point A was measured 2 cm lateral to the tandem and 2 cm superior to the flange (Fig. 3). In Fig. 3, points 1 and 2 represent doses to the vaginal mucosa at the superior end of the applicator. Points 3 and 4 represent doses to the vaginal mucosa at the lower vaginal wall.

**Figure 4:**
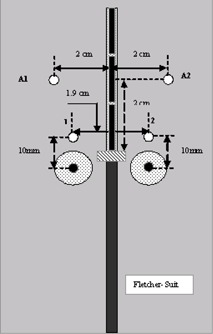
A schematic diagram of the experimental setup for thermoluminescent dosimetry (round symbols) with the Fletcher– Suit applicator.

Similarly, a Solid Water phantom was designed and machined for the Fletcher–Suit applicator such that the measured points were equivalent to those established for the Meigooni applicator. Fig. 4 shows the setup for the Fletcher–Suit applicator.

To determine the dosimetric characteristics of the Meigooni applicator and to compare the results with the characteristics of the Fletcher–Suit applicator, measurements were performed with both applicators using five standard C137s tube sources (Model 6500/6D6C: 3M, St. Paul, MN) in a standard loading pattern—that is, tandem loading (15–10–10) mg Ra eq and ovoid loading (15–15) mg Ra eq. The outside dimensions of the C137s sources were 3.05 mm (diameter) and 20 mm (length) with an active length of 1.4 cm.

The experiments were performed using 1×1×1‐mm LiF TLD chips (TLD‐100: ThermoElectron, Santa Fe, NM). A single TLD was placed in each of the six holes, and to reduce statistical fluctuations in the data, the entire experiment was repeated 5 times. The reported data reflect the average results of 5 separate experiments using an identical standard loading scheme. In addition, doses to the points on the right and left sides of the applicator were averaged to produce the final data presented here.

Table 1 shows the propagation of error for the TLD dosimetry procedures used in these investigations. The TLDs were exposed to radiation, were read using a Harshaw Model 3500 TLD reader, and were annealed using the standard techniques described in detail in previous publications.^(^ ^8^ ^,^ ^9^ ^)^ To demonstrate that the new device can also be used as an alternative to the Fletcher–Suit applicator for patients with cervical cancer, a similar experiment was performed using the Fletcher–Suit applicator with exactly the same loading pattern and the same measuring points. The measured doses at point A and at the superior and lower vaginal walls (points 1, 2, 3, and 4) were compared.

**Table 1:** Propagations of error estimated for the experimental procedures with LiF thermoluminescent dosimeters (TLDs)

	Experimental^a^ (%)		
Component	Type A		Type B
Repetitive measurements	5		
TLD dose calibration uncertainty (including LINAC calibration)			2.0
Exposure timer uncertainty			3.0
Positional uncertainty			2.0
Quadrature sum	5		4.1
Total uncertainty		6.5	

a Type A represents statistical uncertainties, and type B represents systematic uncertainties. All values provided are for 1 *σ*.

### D. Dose calculation using two separate commercially available treatment planning systems

Two separate computer treatment planning systems, Prowess version 3.06 (Prowess, Chico, CA) and BrachyVision version 7.1 (Varian Oncology Systems, Palo Alto, CA) were used for dose calculations at the measuring points. The source data used in the BrachyVision planning system was based on the recent TG‐43 protocol and was obtained from Casal et al.^(10)^ However, the Prowess treatment planning system (version 3.06) is not able to incorporate TG‐43 parameters. Therefore, the C137s data in this system was based on the publication by Meisberger et al.^(11)^

To accurately measure the dose at the points analogous to the TLD experiment, radiographs were taken of the experimental setup, with dummy sources and steel ball bearings representing the locations of the TLDs. The films were then digitized and the parameters were entered into each planning system. The results were recorded and compared to the experimental data.

### E. Dual implants in a T&O with a vaginal cylinder compared with a single implant in the Meigooni applicator

Results from a patient treated using a combination of a T&O and a vaginal cylinder were compared with results of a single implant with the Meigooni applicator. In the former implant type, the total dose at each point was determined by the sum of the dose contributions from each segment of the treatment. In the relevant calculations, we examined two separate loading schemes in the vaginal cylinder: one with the sources fully inserted into the vaginal cylinder, and another with a 3‐cm spacer at the distal end (to reduce the dose to bladder and rectum).

For the implant with Meigooni applicator in the Solid Water setup, we took a set of orthogonal films during which steel ball bearings represented the same critical structure locations and prescription points. The films were then digitized in a commercially available treatment planning system (Prowess), and the calculated dose rates to point A, bladder, and rectum were recorded. A total of five sources were loaded into the tandem with one 2‐cm‐long spacer at the level of the bladder and rectum, and two sources into the ovoids. The actual source strengths were as follows: tandem: 15.65, 11.57, 11.57, 000 (spacer), 6.79, and 8.87 mg Ra eq; ovoid: 8.87 and 8.87 mg Ra eq. Using the same total dose to point A, we compared the dose values determined at each critical structure for each implant procedure.

## III. RESULTS

Table 2 compares the measured and calculated dose values at point A, the superior portion of the vaginal fornices (points 1 and 2), and the lateral vaginal mucosa (points 3 and 4). The measured data presented in Table 2 are the average of the values obtained from 5 separate experiments performed with an identical standard loading scheme. Doses to the points on the right and left sides of the applicator were averaged to achieve the final data presented here. Table 2 also presents the calculations, which were obtained using the BrachyVision treatment planning system.

**Table 2:** A comparison between the dose rates measured using LiF thermoluminescent dosimeters and the dose rates calculated using the BrachyVision planning system (Varian Medical Systems, Palo Alto, CA) for the Meigooni applicator

	Dose rate (cGy/h)		
Position	Measured	Calculated	Difference (%)^a^
A	65.4	60.9	6.8
1,2	266.8	236.4	11.3
3,4	111.4	105.2	5.6

a Values are within 12% for all points.

The results show good agreement (within ±8%) for point A and for the lateral vaginal mucosa. However, differences of up to 12% were found for the superior vaginal fornices. These differences can be attributed to the shortcomings of the algorithm used for calculating the dose at short distances relative to a linear source, to the uncertainty of the source data, and to errors in the reconstruction procedure using the two orthogonal films. The experimental uncertainties were obtained as the standard deviation from the 5 separate measurements.

Similarly, Table 3 shows a comparison between the measured data and the data calculated using the Prowess treatment planning system. Table 4 shows good agreement (within 10%) between the data measured with the Fletcher–Suit applicator and the values calculated using the BrachyVision treatment planning system. The differences in the dose rates to point A from the Meigooni applicator (Table 3) and the Fletcher–Suit applicator (Table 4) can be attributed to the different distances between point A and the ovoid sources because of the geometry of each applicator.

Table 5 shows the dosimetric calculations obtained using the Meigooni applicator for patients with cervical cancer with extension into the lower vagina as compared with the data obtained from a combination of a T&O and a vaginal cylinder implant. The results indicate that the doses to bladder and rectum can be reduced by as much as 37% with the Meigooni applicator, while the dose to the vaginal wall can be increased by about 30%. Furthermore, by implementing a 3‐cm shift in the vaginal cylinder in the dual‐treatment modality combination, dose to the rectum was found to be reduced to about 3.8% relative to the Meigooni applicator (Table 6). However, the bladder dose is still 27% higher than that seen with the Meigooni applicator (Table 6).

**Figure 5:**
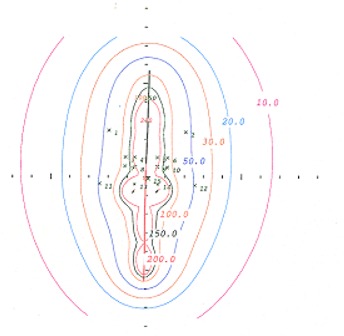
A coronal view of the isodose lines of the Meigooni applicator. These isodose lines were generated using the Prowess treatment planning system (Prowess, Chico, CA).

**Figure 6:**
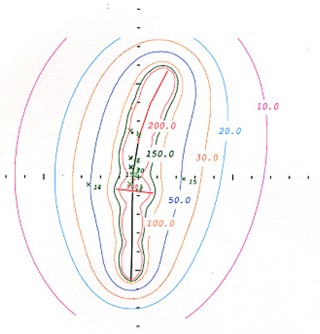
A sagittal view of the isodose lines of the Meigooni applicator. These isodose lines were generated using the Prowess treatment planning system (Prowess, Chico, CA).

Figs. 5 and 6 show the respective isodose distributions in the coronal and sagittal planes for a single implant with the Meigooni applicator, as calculated by the Prowess treatment planning system. These distributions are easily determined and eliminate the difficulty of matching isodose distributions from two separate implants.

**Table 3:** A comparison between the dose rates measured using LiF thermoluminescent dosimeters and the dose rates calculated using the Prowess planning system (Prowess, Chico, CA) for the Meigooni applicator

Position	Dose rate (cGy/h)		
	Measured	Calculated	Difference (%)^a^
A	65.4	63.0	3.7
1,2	266.8	241.0	9.7
3,4	111.4	102.1	8.3

a Values are within 10% for all points.

**Table 4:** A comparison between the dose rates measured using LiF thermoluminescent dosimeters and the dose rates calculated using the BrachyVision planning system (Varian Medical Systems, Palo Alto, CA) for the Fletcher–Suit applicator

Position	Dose rate (cGy/h)		
	Measured	Calculated	Difference (%)^a^
A	71.4	70.9	0.1
1,2	211.8	197.3	6.8

a Values are in good agreement (within 10%) for all points.

**Table 5:** A comparison between treatment with the Meigooni applicator and the values obtained from a combination of the Fletcher–Suit applicator and a vaginal cylinder, for rectal and vaginal doses in a patient with cervical cancer with extension to the lower vagina^a^

Position	Fletcher–Suit with vaginal cylinder (cGy)	Meigooni applicator (cGy)	Difference (%)
A	4000	4000	—
3,4	2500	3570	30.0
Rectum (implant)	4177	3214.4	30.0
Bladder	4539	3298.6	37.6

a Both treatments deliver the same dose to point A, and patients also receive 4500 cGy external‐beam radiotherapy.

**Table 6:** A comparison between treatment with the Meigooni applicator and the values obtained from a combination of the Fletcher–Suit applicator and a vaginal cylinder with a 3‐cm spacer on the top, for rectal and vaginal doses in a patient with cervical cancer with extension to the lower vagina^a^

Position	Fletcher–Suit with vaginal cylinder with 3‐cm spacer at top (cGy)	Meigooni applicator (cGy)	Difference (%)
A	4000	4000	
3,4	2500	3570	30.0
Rectum	3339	3214.4	3.8
Bladder	4539	3298.6	27.4

a Both treatments deliver the same dose to point A, and patients also receive 4500 cGy external‐beam radiotherapy.

## IV. CONCLUSIONS

The Meigooni applicator was designed to treat cervical cancer with extension into the lower vagina. As introduced in this project, the Meigooni applicator provides a method of treating this entire region with one implant instead of with two separate implants as in the current modality. The dose at several points around this applicator has been evaluated and found to be in good agreement (within 10%) with a commercially available treatment planning system and the Fletcher–Suit mini‐ovoid applicator.

The measured dose rates for both applicators were compared with the calculated values from two different treatment‐planning systems to determine the dose delivered to the patient in a planned treatment. The findings of these investigations show that the dose produced at point A with the Meigooni applicator is in good agreement (within 10%) with the dose calculated by BrachyVision equipped with TG‐43 parameters and by Prowess using Meisberger's data. Overall, the Meigooni applicator shows dosimetric characteristics that make it suitable for clinical use. The Meigooni applicator can therefore be used as an alternative to the Fletcher–Suit applicator for patients with cervical cancer.

In a sample patient, treatment using a combination of a T&O and a vaginal cylinder produced bladder and rectal doses that were, respectively, 30% and 37% higher than the doses produced with a single implant using the Meigooni applicator. In addition, the Meigooni applicator, as compared with the Fletcher–Suit applicator, provided 30% more dose to the prescription point inside the vaginal wall. Thus, the Meigooni applicator not only provides the convenience and reduced cost of a single implant, but at the same time delivers 30% more dose to the disease area, while reducing the dose to critical structures by as much as 37%. Moreover, this single device can be used to treat cervical cancer with or without extension into the lower vagina, eliminating the need to purchase two different types of applicators.

## ACKNOWLEDGMENTS

The authors thank Clarissa Wright for her assistance with the dosimetric characteristics of C137s sources.
